# Composites from Recycled HDPE and ZnO Nanopowder with Improved Insulation and Weathering Features for Cable Jacketing Applications

**DOI:** 10.3390/polym17141987

**Published:** 2025-07-20

**Authors:** Alina Ruxandra Caramitu, Magdalena Valentina Lungu, Romeo Cristian Ciobanu, Ioana Ion, Eduard Marius Lungulescu, Gabriela Beatrice Sbarcea, Virgil Emanuel Marinescu, Sebastian Aradoaei, Mihaela Aradoaei, Raducu Machidon

**Affiliations:** 1 National Institute for Research and Development in Electrical Engineering ICPE–CA Bucharest, 030168 Bucharest, Romania; alina.caramitu@icpe-ca.ro (A.R.C.); magdalena.lungu@icpe-ca.ro (M.V.L.); ioana.ion@icpe-ca.ro (I.I.); marius.lungulescu@icpe-ca.ro (E.M.L.); gabriela.sbarcea@icpe-ca.ro (G.B.S.); virgil.marinescu@icpe-ca.ro (V.E.M.); 2 Department of Electrical Measurements and Materials, Gheorghe Asachi Technical University, 700050 Iasi, Romania; arsete@ee.tuiasi.ro (S.A.); mihaela.aradoaei@academic.tuiasi.ro (M.A.); raducu.machidon@gmail.com (R.M.)

**Keywords:** recycled high-density polyethylene, ZnO nanopowder, insulation for cable jacketing applications, moisture resistance, thermal stability

## Abstract

In this study, polymer matrix composites based on high-density polyethylene (HDPE) and recycled HDPE (HDPEr) were reinforced with zinc oxide nanoparticles (ZnO NPs). Four formulations (M1-M4) with HDPE/HDPEr/ZnO NP mass ratios of 50/50/0, 48/47/5, 45/45/10, and 43/42/15 were produced via melt injection molding. Disc-shaped samples (Ø30 ± 0.1 mm × 2 ± 0.1 mm) were evaluated in unaged and aged states (840 h at 100% humidity and 100 °C) using scanning electron microscopy, X-ray diffraction, ultraviolet–visible and Fourier-transform infrared spectroscopy, water absorption, thermal resistance, and mechanical and dielectric testing. Among all composites, M2 showed the best performance, with the highest aging resistance (estimated lifetime of 3891 h in humidity and 2361 h in heat). It also exhibited superior mechanical properties, with the highest indentation hardness, Vickers hardness, and elastic modulus before (0.042 GPa, 3.846 HV, and 0.732 GPa) and after aging under humidity (0.042 GPa, 3.932 HV, 0.706 GPa) and elevated temperature (0.085 GPa, 7.818 HV, 1.871 GPa). Although ZnO NPs slightly reduced electrical resistivity, M2 showed the most stable dielectric properties. In its unaged state, M2 had 22%, 30%, and 3% lower surface resistivity, volume resistivity, and dielectric strength, respectively, than M1 polymer. M2 was identified as the optimal formulation, combining mechanical strength, dielectric stability, and resistance to moisture and heat.

## 1. Introduction

Cable jacketing is regarded as the primary barrier that protects cables from moisture, physical damage, and environmental factors [1,2,3,4]. High-density polyethylene (HDPE) is widely utilized as a cable jacketing material serving to support and insulate overhead transmission lines in urban and rural power grids, as well as in industrial power transmission systems, thereby facilitating the safe and stable flow of electrical energy.

In addition, HDPE is employed as an insulating and protective materials for communication cables, ensuring the stable transmission of communication signals while minimizing interference and leakage. HDPE is characterized by a range of properties that make it highly suitable for cable jacketing applications. It exhibits resistance to a variety of corrosive chemicals, including acids, bases, and solvents. Its mechanical strength and durability provide excellent protection for cables, thereby enhancing their longevity and reliability in both power engineering and telecommunications systems. In mining and construction environments, where heavy equipment and harsh conditions are prevalent, HDPE’s flexibility and capacity to withstand mechanical stress enable it to meet the rigorous demands placed on cable materials [5,6,7]. Properties such as high tensile strength, excellent chemical and moisture resistance, durability under extreme conditions, and ultraviolet (UV) stabilization for outdoor exposure make HDPE a viable material for diverse cable applications. These characteristics contribute to the extended service life and improved operational efficiency of networks, particularly under moist, corrosive, or other harsh environmental conditions [8]. Nevertheless, the relatively low resistance of pure HDPE to thermo-oxidative aging and its flammability represent significant limitations. These drawbacks, although partially mitigated by UV stabilization for outdoor use, restrict the broader applicability of unmodified HDPE in electrical systems [9,10,11].

Significant efforts have been made to enhance the thermal performance, crystallinity, and temperature resistance of HDPE through the incorporation of various inorganic fillers [12,13]. Consequently, composites based on an HDPE polymer matrix and inorganic fillers, including titanium dioxide (TiO_2_), zinc oxide (ZnO), silicon dioxide (SiO_2_), and stannic oxide (SnO_2_) nanopowders, have been fabricated using various techniques, such as melt blending, compression molding, and hot pressing of cast composite solutions [14,15,16,17,18]. The results obtained have demonstrated that these nanocomposites exhibit superior performance compared to the unmodified HDPE polymer. However, limited information is currently available regarding the application of such nanocomposites in the field of electrical insulation. Therefore, recent research has focused on improving the dielectric performance of insulating systems by incorporating inorganic nanoparticles (NPs). Due to their reduced dimensions, these nanoparticles create extensive interfacial areas between the filler and the polymer matrix, which confer distinct advantages, such as a significant reduction in electrical conductivity within a specific concentration range. This behavior indicates the capacity of the polymer matrix to accommodate nanoparticles within its interlamellar regions. Beyond this concentration threshold, additional NPs have been shown to exert minimal influence on the electrical properties of the composite materials [19,20,21,22,23].

In this work, ZnO NPs were employed as a filler due to their semiconducting nature, wide direct bandgap at room temperature (3.37 eV) [24], and excellent electrical properties [2,25], which make them ideal for a variety of practical applications in different engineering and medical fields [25,26]. Moreover, crystalline ZnO nanomaterials are known to exhibit relatively high electron mobility [25,26].

Most studies related to electrical applications have initially focused on low-density polyethylene (LDPE)–ZnO composites for diverse uses, resulting in enhanced mechanical properties, antibacterial performance, and improved resistance to ultraviolet (UV) radiation and corona discharge during aging processes [27,28]. These advancements were subsequently extended to HDPE–ZnO composites, which were synthesized through various methods and processed using different thermoplastic techniques, yielding comparable results [29,30,31]. Typically, ZnO NP loadings in the range of 1–15% wt.% have been investigated, as higher concentrations were found to affect the homogeneity of the composites and necessitate pre-functionalization of the NPs with additional polymers such as silicone [26,32]. However, the outcomes of such approaches have generally been unimpressive, often accompanied by a reduction in insulation performance. It should be noted that a comprehensive investigation of HDPE-based composites containing varying concentrations of ZnO NPs, specifically with regard to aging resistance, is currently lacking, despite its importance for the long-term performance and reliability of cable insulation materials.

The innovation presented in this paper is related to the use of varying concentrations of ZnO NPs to improve the aging resistance of HDPE cable insulation under different environmental conditions, including humidity and elevated temperature. Another notable aspect of this study is the integration of recycled HDPE from Waste Electrical and Electronic Equipment (WEEE) sources to increase the economic benefit and sustainability of the electric cables industry. This was achieved by incorporating a significant proportion of recycled HDPE alongside virgin HDPE, without altering the mechanical and electrical performance of the resulting material. The relevance of using recycled polymers such as HDPE, polypropylene (PP), and their blends as a substitute for virgin polymers in the formulation of advanced materials has been highlighted in the literature [33,34,35,36]. The incorporation of recycled polymers into virgin polymers has been reported to improve mechanical, thermal, and dielectric properties, as also outlined in previous studies [33,34,35,36]. In the present study, the validity of this approach has been demonstrated for cable insulation applications, with improvements observed in both mechanical and electrical properties, as well as enhanced resistance to degradation under humidity and elevated temperature conditions. Additionally, HDPE/HDPEr/ZnO NP composites were produced using melt injection molding a scalable and environmentally friendly method commonly employed for dispersing nanoparticle fillers in polymer nanocomposites [37].

The performance enhancements observed in HDPE/HDPEr/ZnO NP composites are consistent with fundamental scientific principles established in other composite systems [38,39], particularly those involving surface modification, interface engineering, and the incorporation of functional additives. The introduction of ZnO NPs provided polar surface characteristics to the polymer matrix, which facilitated stronger interfacial interactions with the non-polar HDPE through functional surface groups or intrinsic dipole moments [39,40,41]. Furthermore, the interaction between ZnO NPs and the HDPE polymer chains, determined by surface energy effects and the relatively uniform dispersion of the nanoparticles, modified both the bulk and interfacial properties of the composites. These effects improved mechanical and conductive properties, as well as UV resistance of the HDPE/HDPEr/ZnO NP composites compared to unmodified polymer blend.

## 2. Materials and Methods

### 2.1. Materials

Polymer-based materials were prepared using the following raw materials: HDPE–Tipelin 1100 J (Balcar Plast Trade, Bucharest, Romania) with a melt mass flow rate (190 °C/2.16 kg) of 8 g/10 min [42] and a density (23 °C) of 961 kg/m^3^ [43]; recycled HDPE (HDPEr) from WEEE sources prepared by ALL GREEN SRL, Iasi, Romania, and the methodology and characteristics are described in [44,45,46,47]; and ZnO nanopowder (Metall Rare Earth Ltd., Shenzhen, China) with a purity ≥ 99.5%, a maximum particle size of 200 nm, a mean primary particle size of 60 nm, a density (23 °C) of 5.605 g/cm^3^, and a melting point of 1975 °C [24].

### 2.2. Equipment and Methods

#### 2.2.1. Obtaining the Polymer-Based Materials

The raw materials, in a powder form, were mechanically mixed and subsequently passed through a 600 μm sieve. The materials were then introduced into the feed hopper of a Dr. Boy 35A injection machine (Dr. Boy GmbH & Co. KG, Neustadt-Fernthal, Germany), where they were mixed and melted using a melt injection process, with the main operating parameters described in [33]. The temperatures along the screw zones of the injection molding machine were maintained within the range of 150–190 °C, and the applied pressing force ranged between 116 and 120 kN to produce disc-shaped samples measuring 30 ± 0.1 mm in diameter and 2 ± 0.1 mm in thickness.

#### 2.2.2. Characterization Methods

##### SEM Analysis

The microscopic imaging of the structure and morphology of the ZnO NP filler, the HDPE/HDPEr polymer matrix (M1), and the composite materials (M2–M4) was carried out using a field emission scanning electron microscope (FESEM), model Auriga (Carl Zeiss, Oberkochen, Germany), equipped with a focused ion beam (FIB) column, model Canion (Orsay Physics, Fuveau, France). The SEM images were acquired on the cross-sections of the samples using a charge compensation system. Imaging was conducted at an acceleration voltage of 5 kV, utilizing an Everhart–Thornley secondary electron (SE) detector in combination with a Faraday cup.

##### XRD Analysis

Structural characterization was performed at room temperature (RT) using a D8 Discover diffractometer (Bruker AXS GmbH, Karlsruhe, Germany) equipped with a copper (Cu) anode X-ray tube (Cu Kα radiation, wavelength (λ) of 1.5406 Å), operating at a voltage of 40 kV and a current intensity of 40 mA. A scintillation detector was used to collect diffraction patterns over a 2θ range of 10–50°, with a step size of 0.04°, and a measurement time of 2 s per point. The DIFFRAC.EVA v.6.0.0.7 software (Bruker, Karlsruhe, Germany) was employed for XRD data analysis, and the ICDD PDF-2 Release 2022 database (Newtown Square, PA, USA) was used for crystallographic phase identification.

##### UV-Vis Analysis

The absorbance spectra of all samples were recorded at RT in the ultraviolet–visible (UV-Vis) spectral range using a double-beam V-570 spectrophotometer (Jasco Corporation, Tokyo, Japan) equipped with a single monochromator and controlled via the Spectra Manager^TM^ v.1.53.01 software. Measurements were performed over a wavelength range of 800–200 nm, with a fast response, a data interval of 1 nm, a spectral bandwidth of 1.0 nm, and a scanning speed of 100 nm/min. Prior to sample measurements, the spectrophotometer was baseline-corrected using a Spectralon reference tile.

##### FTIR Analysis

Infrared (IR) spectra for all samples were obtained using a Jasco 4200 spectrometer interfaced with a Jasco Pro 470-H Attenuated Total Reflectance (ATR) accessory (Jasco Corporation, Tokyo, Japan) and controlled via the Spectra Manager^TM^ II cross-platform spectroscopy v.2.0 software. Samples were placed on the ATR crystal and measured at RT under controlled pressure. The spectral acquisition parameters included a range of 4000–400 cm^−1^, a resolution of 4 cm^−1^, and 200 scans per spectrum.

##### Dielectric Tests

The dielectric properties, including the real (ε′) and imaginary (ε″) parts of the relative permittivity, as well as the dielectric loss angle tangent (tg δ = ε″/ε′), were determined using broadband dielectric spectroscopy. The measurements were carried out with a Solartron 1260A dielectric spectrometer (Solartron Analytical, Farnborough, UK), employing an alternating electric field with a voltage amplitude of 3 V over a frequency range up to 1 MHz. A measuring electrode with a diameter of 30 mm was used, in accordance with the equations presented in [48]. Dielectric tests were performed both prior to aging and following each aging cycle to estimate the time at which material degradation may occur, as detailed in [49,50].

##### Determination of Resistance to Water Action

To evaluate the effect of water on the obtained materials, the following procedure was employed. Three disc-shaped specimens from each material were immersed in water contained in plastic vessels. The samples were maintained in a submerged state for 168 h, after which they were removed from the water plastic vessels, and then they were gently wiped to remove surface moisture, and placed in a desiccator for 1 h. Subsequently, dielectric property measurements were performed. This process was repeated over five immersion cycles, each lasting 168 h, with measurements conducted at the following cumulative exposure durations: 168 h, 336 h, 504 h, 672 h, and 840 h [50].

##### Determination of Temperature Resistance

The temperature resistance was determined using a Memmert model UF 55 forced convection oven (Memmert GmbH + Co. KG., Schwabach, Germany). Disc-shaped samples were subjected to a temperature of 100 °C over five consecutive cycles, each lasting 168 h, with measurements performed after cumulative thermal exposures of 168 h, 336 h, 504 h, 672 h, and 840 h. After each 168 h cycle, the samples were removed from the oven, placed in a desiccator to cool, and subsequently tested for their optical and dielectric properties. The aging temperature of 100 °C was selected to exceed the maximum operating temperature of the materials, which is about 80 °C in vehicle environments [49].

##### Determination of Volume and Surface Resistivities and Dielectric Strength

The resistivity tests were carried out comparatively for both the initial and aged under humidity and temperature conditions, using a Keithley 6517B/E electrometer (Tektronix/Keithley, Cleveland, OH, USA) in accordance with the procedure described in [51].

The dielectric strength test was performed according to the method outlined in [52], with an applied voltage rate of 2 kV/s.

##### Mechanical Tests

The mechanical properties of disc-shaped samples were evaluated via load-controlled instrumented indentation testing using a Micro-Combi Tester (MCT^2^) equipped with a diamond Berkovich indenter (CSM Instruments, Peseux, Switzerland). Nanoindentation measurements were carried out in accordance with the ISO 14577-1 standard [53] under experimental conditions consistent with those previously reported [33]. A Poisson’s ratio of 0.40 was assumed for all polymer-based samples. Indentation hardness (H_IT_), Vickers hardness (HV), and elastic modulus (E_IT_) were determined using the Oliver and Pharr method [54]. For each sample, five indentations were performed, and results are presented as mean values ± standard deviation (SD).

##### Analysis of the Degree of Water Absorption

Water absorption tests were conducted according to Method 2, point 6.4, of the ISO 62 standard [55] on three samples from each type, with test cycles at 168 h, 336 h, 504 h, 672 h, and 840 h. Samples were initially dried in an oven at 50 ± 2 °C for at least 24 h, cooled, and weighed repeatedly until a constant mass (*m*_1_) within ± 0.1 mg was achieved. Sample masses ranged between 1.2140 g and 1.3069 g. Each sample was then submerged individually in boiling distilled water inside sealed plastic containers. After each cycle, the samples were removed from the water, surface-dried with a dry cloth, and weighed (*m*_2_). Percentage by mass of water absorbed (*c*, in %) for each test sample was calculated using the formulas (1) or (2) provided in [55]:

(1) $$c=\frac{m_{2}{-m}_{1}}{m_{1}}\times 100(\%)$$

(2) $$c=\frac{m_{2}{-m}_{3}}{m_{1}}\times 100(\%)$$

where

*c*—percentage by mass of water absorbed (%);

*m*_1_—mass of the test sample (mg) after initial drying and before immersion in water;

*m*_2_—mass of the test sample (mg) after each cycle of 168 h of immersion in water;

*m*_3_—mass of the test sample (mg) after immersion in water and final drying.

## 3. Results and Discussion

### 3.1. Composition and Labeling of the Studied Materials

Three composite materials of the HDPE/HDPEr/ZnO NP type and a control material based on the HDPE/HDPEr polymer mixture were prepared via injection molding. The composition and labeling of the studied materials are presented in Table 1.

### 3.2. Characterization of the Studied Materials

#### 3.2.1. SEM Analysis

SEM analysis was conducted for the ZnO nanopowder that was used as a filler, the HDPE/HDPEr polymer matrix material labeled as M1, and the polymer composites containing 5, 10, and 15 wt.% ZnO NPs, corresponding to materials labeled M2–M4.

**Figure 1:**
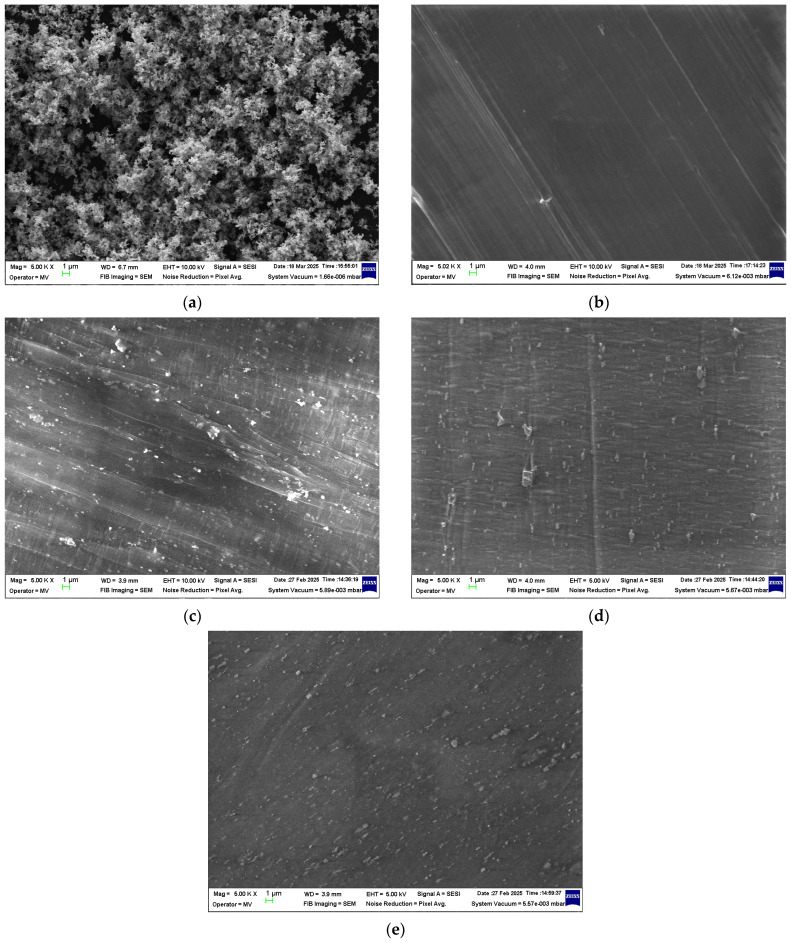
Micrographs at 5000× magnification for (**a**) ZnO nanopowder and the composite samples (**b**) M1, (**c**) M2, (**d**) M3, and (**e**) M4.

The micrographs obtained for these materials at a magnification of 5000× are presented in Figure 1.

Based on the obtained SEM images (Figure 1), the ZnO nanopowder was observed to be relatively well distributed within the polymer matrix across all composite samples (M2–M4). This nanoparticle dispersion suggests effective mixing during melt compounding, which may contribute to the overall structural integrity of the composite. However, slight signs of ZnO NP agglomeration were occasionally detected at higher concentrations, potentially due to van der Waals interactions. These observations are consistent with the literature findings on nanoparticle dispersion in polymer matrices [56,57].

#### 3.2.2. XRD Analysis

**Figure 2:**
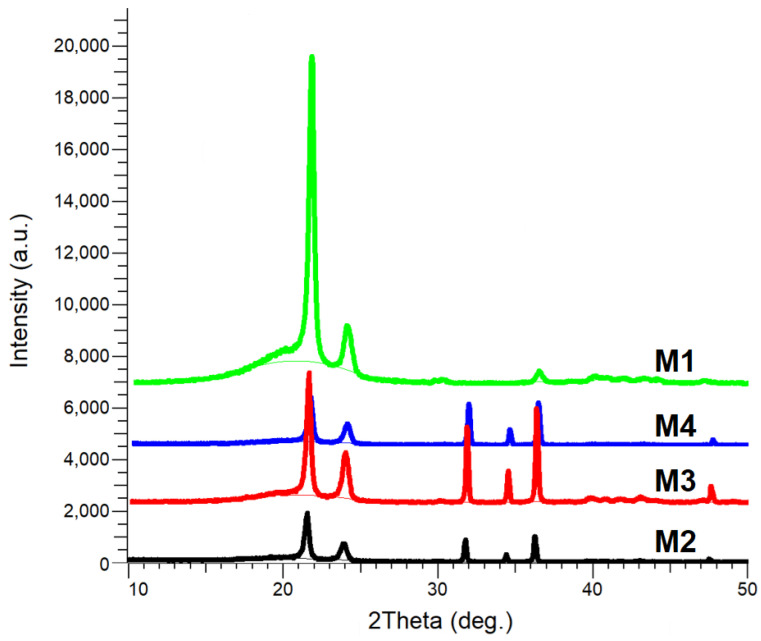
XRD patterns of the samples M1–M4.

The comparative XRD patterns of the samples M1–M4 are presented in Figure 2.

The XRD patterns of all the composite samples (M2–M4) exhibited diffraction peaks corresponding to the hexagonal wurtzite crystalline structure of ZnO (JCPDS Card No. 036-1451), as well as peaks associated with the orthorhombic structure of HDPE (JCPDS Card No. 060-0984), which were also observed in all studied M1–M4 samples.

The diffraction peaks observed at 2θ values of 21.60° and 23.95° were attributed to the (110) and (200) crystallographic planes of HDPE [58]. Peaks detected at 2θ values of 31.77°, 34.42°, and 36.25° were assigned to the (100), (002), and (101) crystallographic planes of ZnO.

No other diffraction peaks were detected in any of the composite samples, indicating the absence of chemical bond formation between the ZnO NPs and the polymer matrix, as also reported in [17].

The lattice parameters and crystallite size of ZnO, determined through Rietveld refinement for the M2-M4 composite samples, are presented in Table 2.

The lattice parameters of the ZnO phase in all composite samples (M2–M4) were found to remain nearly unchanged across the samples, with values of *a* ≅ 3.249 Å and *c* ≅ 5.204 Å, indicating that the crystalline structure of ZnO is preserved regardless of sample composition. However, a progressive decrease in crystallite size (D) was observed, from 144.6 nm in M2 to 132.4 nm in sample M4. This trend may be attributed to the better dispersion of ZnO NPs within the polymer matrix in samples M3 and M4 relative to M2.

Although the ZnO NP content increased from M2 to M4, the reduction in crystallite size may be the result of restricted crystal growth caused by interactions with the polymer matrix, especially in recycled HDPE, where a higher density of interfacial defects or structural constraints may be introduced. Such behavior is commonly reported in polymer nanocomposites, where increased filler content or specific processing conditions can hinder rather than promote crystal growth [16]. The gradual reduction in crystallite size was also accompanied by a decrease in peak intensity of the HDPE/HDPEr polymer matrix, as observed in the XRD patterns (Figure 2).

The incorporation of 5–15 wt.% ZnO NPs into the HDPE/HDPEr polymer matrix was found to disrupt the local crystalline order. This structural alteration was reflected in changes in the intensity of the diffraction peaks, affecting both peak area and peak height. As HDPE exhibits semi-crystalline behavior, its local structural order, such as crystallite size, can be influenced by various factors, including the multiplicity of crystal planes and the structure factor [18].

#### 3.2.3. FTIR Analysis

**Figure 3:**
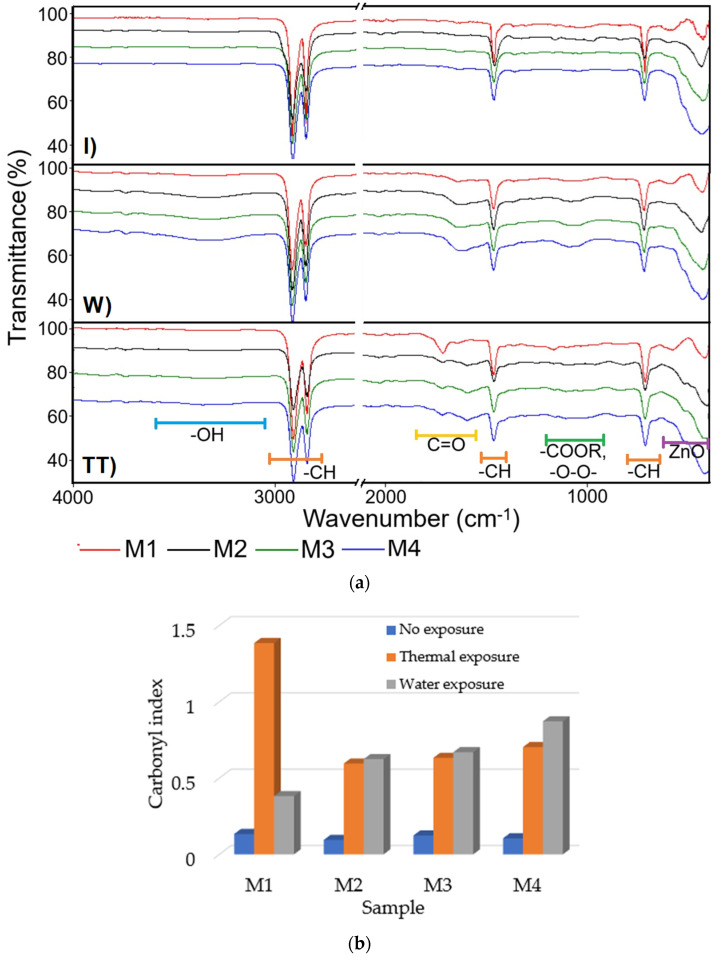
(**a**) ATR/FTIR spectra recorded on the initial samples M1-M4 (I), samples aged under water exposure for 840 h (W), and samples aged under thermal exposure for 840 h at 100 °C (TT). (**b**) Carbonyl index determined from the ATR/FTIR spectra shown in (**a**) for initial and aged samples.

The ATR/FTIR spectra (Figure 3a) illustrate the chemical evolution of HDPE/HDPEr (M1) and composite samples (M2–M4) under accelerated aging conditions relative to unaged state.

In the unaged HDPE/HDPE polymer sample (M1), characteristic absorption bands were observed at 2915 cm^−1^ and 2848 cm^−1^, corresponding to the asymmetric and symmetric stretching vibrations of CH2 groups, along with deformation bands at 1472 cm^−1^ and 1463 cm^−1^, and rocking vibrations around 730 cm^−1^ and 720 cm^−1^, indicating a stable, non-degraded polyethylene backbone [59]. After 840 h of water immersion, a weak carbonyl band emerged at 1715 cm^−1^, accompanied by a broad shoulder around 3400 cm^−1^, suggesting the initiation of oxidative degradation via hydrolytic attack and water uptake. Thermal aging at 100 °C for 840 h resulted in a pronounced carbonyl band, along with new absorption peaks near 1100 cm^−1^, attributed to ester and peroxide groups, which are indicative of chain scission and advanced oxidative degradation [59].

Incorporation of 15 wt.% ZnO NPs into the HDPE/HDPEr polymer matrix significantly altered the degradation response. For the water-aged composite sample, the intensity of the carbonyl peak was notably reduced compared to the unfilled polymer (M1), and a band below 600 cm^−1^, associated with Zn–O vibrations, was observed, confirming the presence of ZnO NPs and their role in mitigating oxidative processes. In the thermally aged composites, evidence of oxidation was still present, but the carbonyl peak intensity was lower than that of the unfilled thermally aged polymer blend (M1), indicating partial protection provided by ZnO under severe thermal stress.

Thermal aging at 100 °C for 840 h gives a stronger carbonyl band, together with new peaks in the 1100 cm^−1^ range, which are due to ester and peroxide groups, which are excellent indicators of chain scission and oxidative degradation. Incorporation of 15 wt.% ZnO NPs into the HDPE/HDPEr polymer matrix radically alters this response. For the water-aged samples, the carbonyl peak is greatly diminished compared to the unfilled polymer blend (M1), and the presence of a band below 600 cm^−1^ may be due to Zn–O vibrations, confirming the presence of ZnO NPs and their role in oxidation prevention. The composites thermally aged at 100 °C continue to display evidence of oxidation, although with less carbonyl intensity than the unfilled thermally aged polymer blend (M1), indicative of the partial protection provided by ZnO under severe thermal stress.

The carbonyl index (CI), an indicator of polymer oxidation, is determined by the ratio of the IR absorbance of carbonyl groups (approximately 1700 cm^−1^) to that of a stable reference peak. An increase in CI reflects progressive degradation caused by exposure to heat, UV radiation, or oxygen [60]. The CI was calculated using Equation (3) [61].

(3) $$CI=\frac{A_{1780-1560}}{A_{1480-1420}}$$

where A_1780–1560_ represents the integrated area of the carbonyl absorption band (1780–1560 cm^−1^), and A_1480–1420_ corresponds to the area of the methylene C–H bending band (1480–1420 cm^−1^), which was used as an internal reference.

In Figure 3b, the influence of exposure conditions on the evolution of the carbonyl index is presented, calculated as the ratio between the integrated areas of the carbonyl peak (arising from ketones, aldehydes, carboxylic acids, etc.) and the C–H peak, using Equation (3) [61]. Thermal aging at 100 °C for 840 h resulted in a significantly more intense carbonyl band, along with the emergence of new peaks around 1100 cm^−1^, attributed to ester and peroxide groups, which are well-established indicators of chain scission and oxidative degradation [59].

The incorporation of ZnO NPs into the polymer matrix was found to significantly modify the thermal oxidation response, particularly after aging at 100 °C. Under these conditions, the carbonyl index decreased notably relative to the unfilled polymer blend (M1), from 1.3853 in sample M1 to 0.5956, 0.6330, and 0.7034 for samples M2, M3, and M4. This reduction indicates a stabilizing effect provided by ZnO NPs, likely due to their free radical-trapping capacity. Furthermore, characteristic Zn–O vibrational bands were detected below 600 cm^−1^ in the ATR/FTIR spectra, confirming the presence of ZnO NPs.

After 840 h of immersion in water, the carbonyl index was observed to increase with the ZnO NP content, rising from 0.3822 in M1 to 0.8721 in M4. This increase suggests the onset of oxidative degradation, potentially facilitated by hydrolytic activity or enhanced water uptake. Additionally, spectral overlap between hydroxyl and carbonyl absorption bands may have contributed to this trend. These findings are consistent with previous studies [15,62,63], which have reported that ZnO NPs serve as effective thermal and UV stabilizers through radical trapping and the formation of a physical barrier to oxygen and moisture diffusion.

The carbonyl index recorded for the unexposed samples M1-M4, ranging between 0.0954 and 0.1342, is attributed to oxidation that occurred during melt processing in the extruder, in the absence of stabilizing antioxidants.

#### 3.2.4. UV-Vis Analysis

Considering the desired application for these materials, resistance to UV radiation is a critical factor. It is well known that most polymers are susceptible to degradation under UV exposure [14,62]. However, the incorporation of ZnO NPs into the polyethylene matrix has the role to protect the composite from the harmful effects of UV radiation.

**Figure 4:**
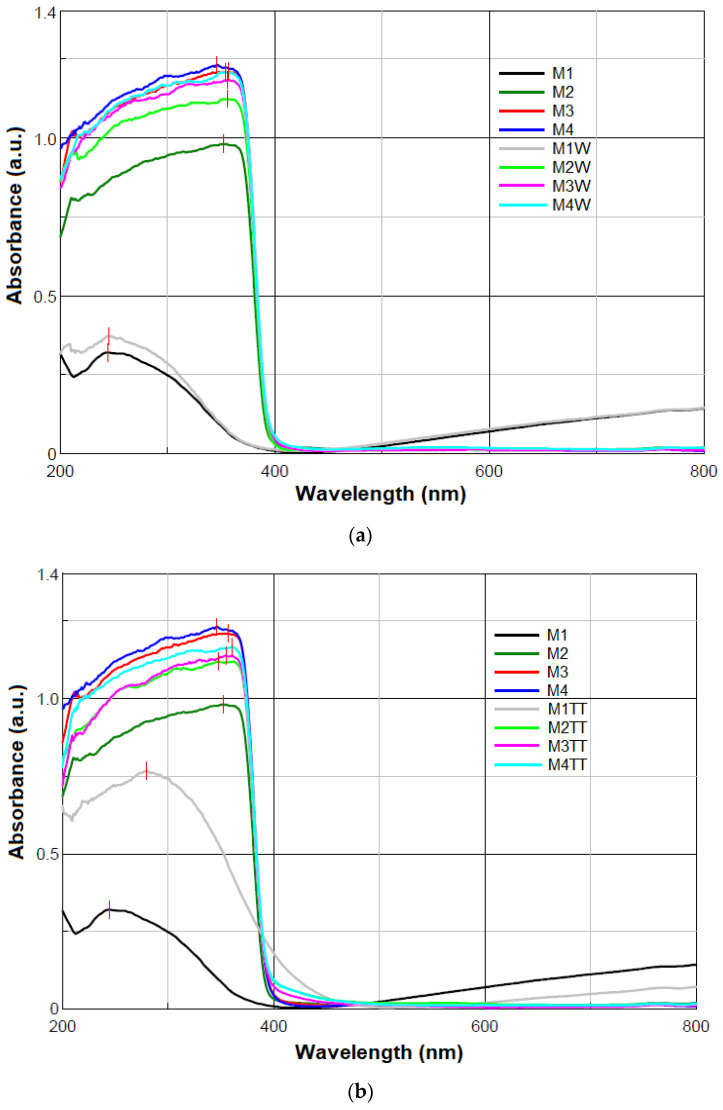
UV-Vis absorbance spectra of the samples: (**a**) M1–M4 and M1W–M4W and (**b**) M1–M4 and M1TT–M4TT.

The UV-Vis absorbance spectra for the unaged and aged samples, which were exposed to humidity and elevated temperature (100 °C) for 840 h, are comparatively presented in Figure 4. The corresponding maximum absorbance values of the studied materials are summarized in Table 3.

The polymer blend sample without ZnO NPs (M1) exhibited a UV absorption peak at the maximum wavelength (λ_max_) of 244 nm, corresponding to electronic transitions within the polymer backbone. Upon the incorporation of ZnO NPs in the HDPE/HDPEr matrix (M2-M4), a red shift in the maximum absorbance was observed, with λ_max_ values ranging from 346 nm to 357 nm in the unaged state and 354 nm to 361 nm after artificial aging (840 h at 100% humidity and 100 °C).

The UV-Vis spectra of all composites (Figure 4) showed similar profiles, characterized by strong absorption in the UV region, consistent with the bandgap absorption of ZnO [24,64]. Despite the relatively high ZnO content, only minor spectral differences were noted among M2-M4 samples, likely due to the encapsulation of ZnO within the polymer matrix, which limits its direct optical contribution.

All M2-M4 samples exhibited high absorbance (0.982–1.168 a.u.) in the UV region (200–400 nm), followed by a sharp decline, with minimal absorption in the visible region (400–800 nm), in agreement with the typical optical response of ZnO-based nanocomposites [24,64]. The observed peak shifts and enhanced absorbance confirm the presence and UV-active role of ZnO, while slight variations among M2–M4 samples may reflect differences in NP dispersion, concentration, or interfacial interactions with the polymer matrix.

The white ZnO nanopowder used as a filler in the HDPE/HDPEr polymer matrix exhibits two characteristic absorption peaks at 359 nm and 307 nm, as previously reported [24]. However, when 5–15 wt.% ZnO NPs were incorporated into the polymer matrix, only the peak near 359 nm was observed in the M2-M4 composites, both in the unaged state and after artificial aging under humidity and temperature conditions. The absence of the 307 nm peak may be attributed to the encapsulation of ZnO NPs within the polymer matrix, which suppresses surface-related transitions, modifies the local dielectric environment, and introduces scattering effects that diminish the visibility of this higher-energy absorption feature. Similar observations have been reported in the literature, where ZnO NPs embedded in polymer matrices display primarily the bandgap absorption peak, while surface-related features are attenuated [64,65].

Environmental factors (temperature and moisture) were found to induce spectral changes in M1-M4 samples, with temperature having a more pronounced effect. A slight increase in λ_max_ from 244 nm to 245 nm and absorbance from 0.321 a.u. to 0.372 a.u. was observed for the M1W sample, indicating minor oxidative degradation induced by moisture. In the M2W-M4W samples, λ_max_ values were in the 354–356 nm range, suggesting that ZnO retained its optical stability under humid conditions. The UV-Vis spectra of the M3 and M4 samples after 840 h of exposure to moisture showed negligible changes, with slightly increased absorbance relative to M2. This increase may be attributed to modifications in ZnO surface states or good ZnO NP dispersion due to hydrolytic interactions.

A strong red shift from 244 nm to 279 nm, accompanied by a significant increase in absorbance from 0.321 a.u. to 0.769 a.u. (Table 3), was observed for the M1TT sample. This 35 nm shift indicates the oxidative degradation of the polymer matrix after prolonged exposure (840 h) to 100 °C. During this process, oxygen-containing functional groups, such as carbonyl (C=O), hydroxyl (–OH), and carboxyl (–COOH), were formed due to polymer chain scission and oxidation. These groups, confirmed by FTIR analysis (Figure 3), act as chromophores, resulting in increased UV absorption and optical density [66].

The λ_max_ values of the M2TT-M4TT samples exhibited slight shifts compared to M2–M4 samples, indicating that the bandgap absorption of ZnO was preserved after thermal aging. The red shift observed in M4TT, from 346 nm to 361 nm, may be attributed to interfacial interactions between ZnO NPs and the polymer matrix affecting light absorption. Furthermore, the changes in UV-Vis absorbance were likely caused by oxidative degradation within the polymer matrix, altering the local dielectric environment. This behavior aligns with the reported polyethylene degradation mechanisms, where thermal oxidation induces chain scission and crosslinking, leading to modified optical properties [58,66,67,68].

#### 3.2.5. Dielectric Tests

Dielectric tests were performed comparatively on the composite materials and the reference polymeric material (HDPE/HDPEr) to evaluate the onset of material degradation. In this aim, all samples were subjected to five cycles of 7 days, each under controlled humidity and temperature conditions. After each cycle, dielectric properties were measured and compared to those of the reference sample (M1).

The onset of degradation was identified by an increase in the dielectric loss tangent (tg δ) exceeding 30% relative to its initial value.

**Figure 5:**
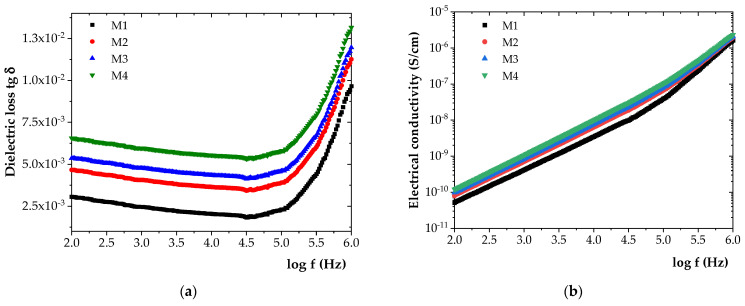
Frequency variation in (**a**) tg δ and (**b**) σ for the initial samples.

The initial values of the dielectric loss tangent (tg δ) and electrical conductivity (σ) for all investigated samples are presented in Figure 5. The measured values of tg δ and σ for samples M1-M4, recorded at five representative frequencies (0.1 kHz, 1 kHz, 10 kHz, 100 kHz, and 1000 kHz), are summarized in Table 4.

As shown in Figure 5, the incorporation of increasing amounts of ZnO NPs into the HDPE/HDPEr matrix led to progressive increases in both tg δ and electrical conductivity (σ). Compared to M1, tg δ increased by approximately 35% for M2, 43% for M3, and 54% for M4. Similarly, σ increased by approximately 22% for M2, 30% for M3, and 39% for M4.

An increase in tg δ was found to indicate enhanced dielectric losses and, implicitly, a reduction in the insulating performance of the material. Based on the experimental data, only slight variations in dielectric properties were observed among the materials, which can be attributed to the dominant presence of the HDPE/HDPEr polymer blend.

##### Variation in Dielectric Properties with Humidity

**Figure 6:**
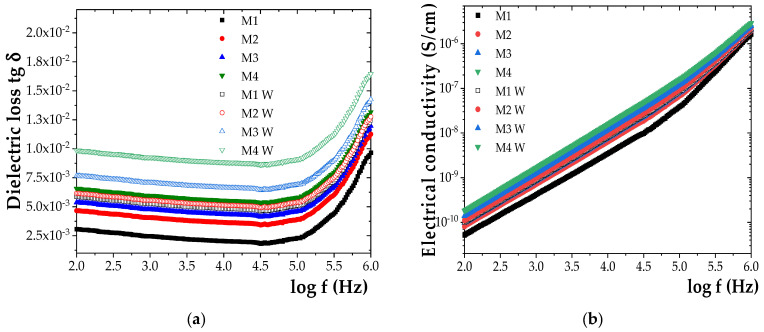
Frequency dependence of (**a**) dielectric loss tangent (tg δ) and (**b**) electrical conductivity (σ) for the initial samples M1-M4 and samples M1W-M4W aged for 840 h under humidity.

Experimental data showing the effect of humidity on the dielectric properties are presented in Figure 6. For improved visualization, only the values corresponding to the initial state and those after the maximum exposure duration (840 h) were included for comparison.

A clear trend of increasing tg δ and σ values with prolonged exposure to humidity was observed for all investigated samples (Figure 6). After 840 h of exposure, tg δ increased by approximately 48% for M1, 51% for M2, 60% for M3, and 69% for M4. A similar trend was noted for σ, which increased by 31% for M1, 35% for M2, 45% for M3, and 54% for M4. These findings indicate a progressive degradation of dielectric performance under humid conditions, amplified by the ZnO NP content.

##### Determination of the Remaining Lifetime Under Humidity Conditions

The remaining lifetime of the materials investigated in this study is a critical parameter for equipment incorporating insulated cables, as it estimates the duration before material degradation reaches a level requiring replacement.

Timely replacement is essential to ensure proper equipment function and to prevent potential damage. Based on the experimental results obtained from humidity resistance testing, the remaining lifetime until significant degradation was determined for each composite material [69].

The variation in electrical resistivity at 1 MHz, measured at different humidity exposure durations, is shown in Table 5. The critical resistivity was defined as 30% of the initial electrical resistivity value at 1 MHz, and was used as the degradation threshold.

The calculation method was based on the procedure described in [70] and involved plotting the electrical resistivity values corresponding to each aging period (as presented in Table 5).

From the linear equation derived from the trend line, the total lifetime (x) of the material was determined. By subtracting the 840 h of artificial aging from the calculated lifetime, the remaining lifetime was obtained.

The first-degree equations describing the trend of the variation curves were extracted, and the corresponding values presented in Table 6 were obtained. The remaining operating lifetime under normal conditions was calculated by subtracting the 840 h of accelerated aging from the total lifetime values. Based on these results, the composite material exhibiting the fastest degradation rate was also identified.

The polymer blend (M1) was found to be the least resistant to moisture. Based on the data presented in Table 6, the composite material M4 exhibited the lowest moisture resistance among the ZnO-reinforced composites, as evidenced by the shortest remaining lifetime until total degradation (2181 h). In contrast, M2 demonstrated the highest resistance to moisture, with a remaining lifetime of 3891 h, followed by M3 with 2814 h, and M4 with 2181 h. These values were determined under accelerated aging conditions and reflect the relative stability of each composite material when exposed to moisture.

This behavior can be attributed to the mechanical interactions that occurred during the melt-processing of the polymer/ZnO nanocomposite materials. Due to the strong polarization of the ZnO NPs, slight repulsive forces were induced within the polymer matrix. These forces resulted in the formation of micron-sized voids, which were only partially filled by ZnO NPs. As observed from the experimental results, when comparing the remaining lifetime of the composites to that of the HDPE-based material (M1), the lifetime was found to increase by approximately 94% for M2, 40% for M3, and 9% for M4.

This analysis indicates that incorporating ZnO NP concentrations greater than 5 wt.% does not lead to further improvement in resistance to humidity. Moreover, it results in a decrease in the composite’s lifetime. Thus, the optimal ZnO NP content for enhancing humidity resistance was identified as 5 wt.%, corresponding to the M2 composite.

Based on the performed tests, the longest remaining lifetime was recorded for M2, followed by M3, M4, and M1. Accordingly, the M2 composite with an HDPE/HDPEr/ZnO NP mass ratio of 48/47/5 demonstrated the highest resistance to humidity. Therefore, M2 is recommended as a suitable material for external insulation in cables operating in high-humidity environments.

##### Variation in Dielectric Properties with Temperature

**Figure 7:**
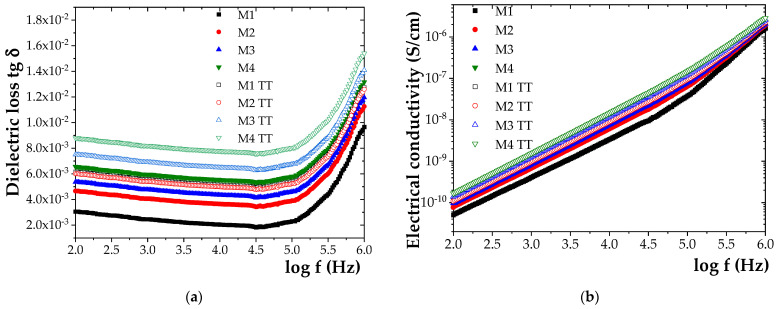
Frequency dependence of (**a**) dielectric loss tangent (tg δ) and (**b**) electrical conductivity (σ) for initial samples M1–M4 and samples M1W-M4W aged for 840 h at 100 °C.

The experimental values are presented in Figure 7, where the initial values and those measured after 840 h of thermal exposure at 100 °C are shown comparatively. The data were represented separately to ensure clarity, as displaying all curves on a single graph rendered the figure unreadable.

Based on the results, it was observed that, after 840 h of exposure to elevated temperature, the loss tangent (tg δ) increased by approximately 52% for M1, 50% for M2, 60% for M3, and 66% for M4. A similar trend was observed for electrical conductivity (σ), which increased by approximately 38% for M1, 37% for M2, 47% for M3, and 53% for M4.

##### Determination of Remaining Lifetime Under Temperature Conditions

From the experimental results obtained during the tests evaluating resistance to 100 °C temperature exposure, the remaining lifetime was determined for each composite material. The calculation method described in [70] was applied, which involves plotting the electrical resistivity values for each aging period (see Table 7 and the description in Section Determination of the Remaining Lifetime Under Humidity Conditions).

The variation in electrical resistivity of the composites at a frequency of 1 MHz for each exposure time to temperature is presented in Table 8. The critical resistivity was defined as 30% of the initial electrical resistivity value at 1 MHz.

First-degree equations describing the trend of the variation curves were derived, from which the data in Table 8 were obtained. The remaining service life under normal operating conditions was calculated by subtracting the 840 h of accelerated aging from the total time extrapolated. Additionally, these data allowed the identification of the composite exhibiting the fastest degradation. Such information is essential for industrial applications to enable the timely replacement of composites subjected to excessive temperature conditions.

It was found that the composite sample M2 exhibited the highest temperature resistance among the analyzed materials. This is evidenced by the longest remaining lifetime until degradation, measured at 2361 h. The remaining lifetimes were 1702 h for M3, 1651 h for M4, and 1165 h for the reference sample (M1).

The polymer blend M1 was observed to degrade most rapidly under thermal exposure, as indicated by its shortest remaining lifetime. Compared to M1, the remaining lifetimes of the composites aged at elevated temperature increased by approximately 51% for M2, 32% for M3, and 29% for M4. Therefore, the composite M2 demonstrated the greatest thermal resistance and is recommended for use as external insulation in cables operating under excessive temperature conditions.

Based on the dielectric test results, the composite material M2 was qualified as exhibiting the best resistance to degradation relative to the reference polymer M1 and the other composites, supporting its recommendation for application as external insulation in electrical cables subjected to harsh humidity and temperature environments.

##### Determination of Surface and Volume Resistivity and Dielectric Strength

The external insulations (sheaths) of cables fabricated from the M2 composite require not only good mechanical properties but also high electrical insulation properties to avoid major safety hazards [71].

1. Determination of surface and volume resistivities

**Figure 8:**
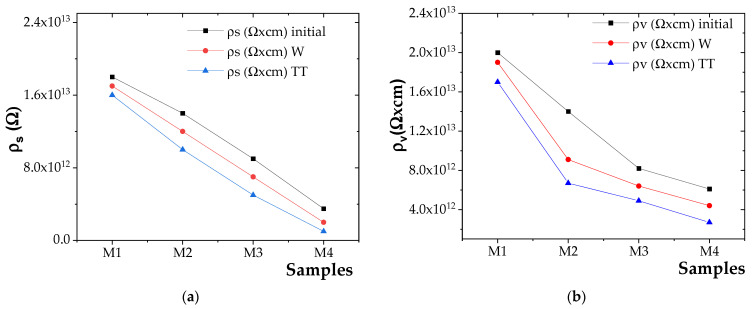
(**a**) ρ_s_ variation for initial and aged samples and (**b**) ρ_v_ variation for initial and aged samples.

The experimental results for surface resistivity (ρ_s_) and volume resistivity (ρ_v_) are presented in Figure 8a for ρ_s_ and Figure 8b for ρ_v_.

Based on the obtained results, it was observed that both the surface resistivity (ρ_s_) and volume resistivity (ρ_v_) varied inversely with the concentration of ZnO NPs [72]. The incorporation of ZnO NPs filler was found to inhibit the movement of charge carriers due to the interfacial effects at the polymer–filler boundary, thereby increasing the material’s resistance to current flow and resulting in higher ρ_s_ and ρ_v_ values [73].

However, at higher ZnO NP concentrations and lower NP size, agglomeration was observed to become more pronounced, which diminishes the interfacial effect and facilitates increased charge carrier mobility [74]. This led to enhanced electrical conductivity and, consequently, a reduction in ρ_s_ and ρ_v_ values [56].

The highest surface and volume resistivities were recorded for the M2 composite compared to M3 and M4 composites. Although ZnO NPs slightly reduced electrical resistivity, M2 showed the most stable dielectric properties. In its unaged state, M2 composite had approximately 22%, 30%, and 3% lower surface resistivity, volume resistivity, and dielectric strength, respectively, than the reference polymer blend (M1).

2. Determination of dielectric strength

**Figure 9:**
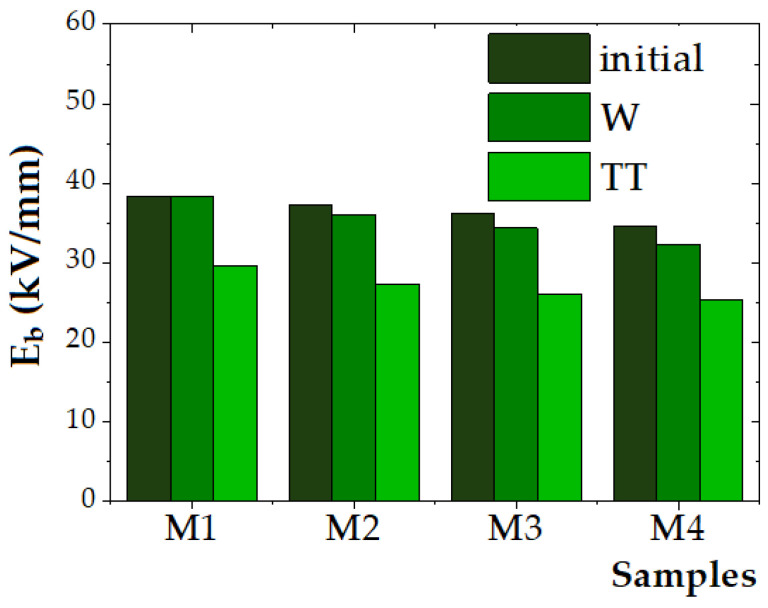
Dielectric strength (E_b_) of the initial and aged samples.

The experimental values obtained for the dielectric strength (E_b_) of the initial and aged samples are presented in Figure 9.

The reference polymer blend sample (M1) exhibited the highest dielectric strength of 38.33 kV/mm, while the dielectric strength of the composite samples (M2–M4) decreased from 37.33 kV/mm to 34.67 kV/mm with an increase in the ZnO NP content. For the unaged samples, the dielectric strength decreased by approximately 2.6% for M2 (5 wt.% ZnO), 5.6% for M3 (10 wt.% ZnO), and 9.6% for M4 (15 wt.% ZnO), compared to the M1 polymer material. This trend aligns with the findings reported by Zare et al. [57] and Mansour et al. [75], who observed that the addition of ZnO NPs beyond an optimal concentration leads to particle agglomeration, which introduces localized electric field enhancements and structural defect sites in the polymer matrix that facilitate charge accumulation, thus reducing dielectric breakdown strength.

Mansour et al. [75] disclosed findings on the dielectric strength of nanocomposites based on a pure HDPE matrix reinforced with varying weight percentages of ZnO nanoparticles (0.3%, 0.5%, 1%, 3%, and 5%) with a particle size in the range of 40–75 nm. No significant improvement was detected up to 0.5% ZnO, whereas at 1%, the dielectric strength increased by 17 ± 3.1% compared to pure HDPE, attributed to enhanced charge trapping and improved electric stress distribution. A slight decrease was noted at 3%, and at 5% ZnO, as the dielectric strength dropped below that of pure HDPE, possibly due to nanoparticle agglomeration. The optimal enhancement was observed at 1% ZnO NPs. The variation in dielectric strength with filler concentration confirmed the role of interfacial area in electric stress distribution and the path length during DC breakdown.

In this study, after exposure to humidity (840 h), only minor reductions in dielectric strength were observed, with decreases ranging from 0% (M1) to 3.4% (M2), 5.2% (M3), and 6.7% (M4), indicating that the composites retain satisfactory dielectric performance under moist conditions. This observation is consistent with studies by Rohani et al. [76], who reported that the incorporation of ZnO NPs to polymer matrices can improve water resistance due to their surface activity and ability to form interfacial hydrogen bonding with the polymer matrix, thereby mitigating water-induced conductivity increases.

Thermal aging at 100 °C for 840 h caused a more pronounced degradation in dielectric strength, with reductions of 22.6% for M1, 26.8% for M2, 27.8% for M3, and 26.9% for M4. This finding supports the well-established understanding that polyethylene-based materials undergo thermo-oxidative degradation under prolonged thermal stress, as reported by Ni et al. [10] and Zhang et al. [77]. The formation of polar oxygenated species such as carbonyl and hydroxyl groups contributes to increased conductivity and premature dielectric breakdown. Notably, M2 (5 wt.% ZnO NPs) exhibited the smallest decrease in dielectric strength (26.8%), suggesting that moderate ZnO NP loading may provide a stabilizing effect. This could be attributed to the role of ZnO as a heat sink, which reduces chain scission and delays oxidation, as proposed by Elsadd et al. [32].

Nonetheless, while ZnO NPs enhance environmental resistance, their concentration must be optimized to preserve dielectric integrity, as excess loading promotes defect formation and lowers breakdown strength. To enhance dielectric strength in polymer nanocomposites, the difference in relative permittivity and electrical conductivity between the nanofiller and base polymer matrix has to be minimized [78].

#### 3.2.6. Mechanical Properties

The experimental data for the unaged and aged samples, which were exposed to humidity and elevated temperature (100 °C) for 840 h, are presented in Table 9.

The variations in mechanical properties of the unaged and aged composite samples relative to M1 are provided in Table 10.

The Shore D hardness and elastic modulus of polypropylene/high-density polyethylene (PP/HDPE) blends and their ZnO nanopowder-reinforced composites (an NP size of 20–90 nm) were reported by Ban Jawad Kadhim et al. [79]. An increase in the HDPE content (10–50 wt.%) led to a reduction in hardness from 65 to 50.6 Shore D and in elastic modulus from 0.32 GPa to 0.13 GPa. In contrast, ZnO NP incorporation enhanced both mechanical properties. For the 90/10 PP/HDPE blend, hardness increased from 58 to 62.6 Shore D and elastic modulus from 0.32 GPa to 0.71 GPa with 1–3 wt.% ZnO. Similarly, for the 80/20 blend, hardness increased from 55 to 61.3 Shore D and elastic modulus from 0.25 GPa to 0.76 GPa. These improvements were attributed to the inherent stiffness of ZnO and its ability to occupy interstitial spaces between polymer chains. Additionally, surface hardness was influenced by the homogeneity and smoothness of the materials [79].

Unlike conventional polymers, the mechanical properties of polymer composites can be tailored through the selection of constituent components, their concentrations, the geometry and orientation of the fillers, and the blending methods and processing conditions employed. These properties are significantly influenced by the strength and stiffness of the fillers, which are typically greater than those of the polymer matrices [56].

In this study, the superior mechanical performance of the M2 composite (5 wt.% ZnO NPs), compared to both the unfilled polymer (M1) and composites with higher ZnO NP contents (M3 and M4, with 10 wt.% and 15 wt.%, respectively), can be attributed to the relatively well-dispersed ZnO NPs and stronger interfacial interactions between the nanoparticle filler and polymer matrix [80]. At higher concentrations, ZnO NPs tend to agglomerate due to van der Waals interactions. These agglomerates act as stress concentrators, promoting microcrack initiation and compromising mechanical integrity especially under harsh environmental conditions [56,57].

For unaged samples, the highest values of HIT (0.042 ± 0.004 GPa), HV (3.846 ± 0.388), and EIT (0.732 ± 0.030 GPa) were exhibited by M2. Similar trends were observed for water-aged samples, with M2W maintaining superior values among all tested groups. After thermal aging, the highest mechanical properties were also demonstrated by M2TT, with an HIT of 0.085 ± 0.005 GPa, HV of 7.918 ± 0.475, and EIT of 1.871 ± 0.152 GPa.

These results confirm that, at 5 wt.% ZnO NPs, interfacial adhesion between the filler and polymer matrix is optimized, enhancing stiffness and strength by restricting polymer chain mobility [56,57]. At higher ZnO NP filler contents, reduced effective surface area and matrix oversaturation compromise the efficiency of the reinforcement effect [57].

#### 3.2.7. Analysis of the Degree of Water Absorption

The amount of water absorbed by the HDPE/HDPEr/ZnO composite materials (M2–M4) was analyzed by comparison with that absorbed by the polymer matrix (M1). The experimental results are presented in Table 11.

Water absorption within the composite samples was attributed to the mechanical interactions between the polymer and the ZnO NP filler. These interactions arise from the strong polarization of the ZnO NP filler, which induces slight repulsive forces. As a result, micron-scale voids are formed between particles, resulting in the non-uniform dissociation of the filler particles within the polymer matrix.

Based on the water absorption tests conducted over 840 h under 100% humidity, the lowest water uptake was observed for the M2 composite, followed by M1, M3, and M4, respectively. These findings are consistent with the results obtained from both the remaining service life evaluation under aging conditions (humidity and temperature) and the mechanical property tests.

Therefore, from the perspective of water absorption, the M2 material is recommended for use as the outer sheath in cable insulation applications intended for operation under excessive humidity conditions.

## 4. Conclusions

In this study, polymer composites based on a virgin and recycled high-density polyethylene (HDPE/HDPEr) matrix reinforced with ZnO nanoparticles (NPs) were developed via melt injection molding. The aim was to produce advanced materials with enhanced performance characteristics suitable for electrical cable insulation. Four material formulations (M1-M4), varying in the ZnO content (0–15 wt.%), were synthesized. The HDPE/HDPEr/ZnO NP mass ratios of the synthesized materials were 50/50/0 for M1, 48/47/5 for M2, 45/45/10 for M3, and 43/42/15 for M4.

The obtained disc-shaped samples measuring 30 ± 0.1 mm in diameter and 2 ± 0.1 mm in thickness were subjected to comprehensive characterization, including morphological (SEM), structural (XRD, FTIR), optical (UV-Vis), mechanical, dielectric, and water absorption analyses. These tests were performed on both unaged and artificially aged samples (840 h at 100% relative humidity and 100 °C, respectively).

The SEM analyses confirmed relatively well-dispersed ZnO NPs across all composites, with ZnO acting as a partial thermal stabilizer in aged samples by trapping free radicals and impeding oxygen and moisture diffusion. The data also revealed that water absorption and mechanical degradation followed a consistent trend across both aging conditions, providing insight into the degradation timeline of the insulation materials and supporting predictive maintenance strategies for electrical equipment.

Among the studied formulations, the M2 composite (48 wt.% HDPE, 47 wt.% HDPEr, and 5 wt.% ZnO NPs) exhibited the most favorable properties. Compared to the unfilled matrix (M1), M2 demonstrated an approximate 15% increase in mechanical performance and a 10% improvement in dielectric strength, along with superior resistance to thermal and moisture-induced aging. These results suggest that M2 is the most promising candidate for cable insulation applications in demanding environmental conditions.

Further investigation is needed to better understand the mechanisms behind the superior performance of the M2 formulation, particularly the role of nanoparticle dispersion and interfacial interactions at low filler concentrations.

In summary, the addition of ZnO filler in HDPE/HDPEr matrices increases the remaining lifetime for samples exposed to humidity and temperature, as well as mechanical resistance. However, the addition of fillers leads to a slight decrease in the volume and surface resistivities, as well as the breakdown voltage, but the significant advantage from enhanced mechanical and environmental resistance can entirely offset the minor reduction in electrical performance, which stays anyway within the set limits for cable jacketing applications.

## Figures and Tables

**Table 1:** Composition and labeling of the studied materials.

Samples	Component Concentration		
	HDPE (wt.%)	HDPEr (wt.%)	ZnO NPs (wt.%)
M1	50	50	0
M2	48	47	5
M3	45	45	10
M4	43	42	15
Samples	Aging Conditions		
MxW (x = 1, … 4)	840 h in water (100% humidity)		
MxTT (x = 1, … 4)	840 h of thermal treatment at 100 °C		

**Table 2:** Structural properties of the composite samples (M2–M4).

Samples	Crystallographic Phase	Lattice Parameters		Crystallite Size D (nm)
		a (Å)	c (Å)	
M2	ZnO	3.249	5.204	144.6
M3	ZnO	3.249	5.205	138.7
M4	ZnO	3.248	5.203	132.4

**Table 3:** Maximum absorbance of the studied materials.

Maximum Absorbance	Samples											
	M1	M2	M3	M4	M1W	M2W	M3W	M4W	M1TT	M2TT	M3TT	M4TT
λ_max_ (nm)	244	352	357	346	245	356	356	354	279	348	355	361
Abs (a.u.)	0.321	0.982	1.211	1.231	0.372	1.125	1.184	1.209	0.769	1.120	1.139	1.168

**Table 4:** tg δ and σ for frequencies in the range of 0.1 kHz–1 MHz.

Frequency (kHz)/Samples	0.1	1	10	100	1000	0.1	1	10	100	1000
	tg δ × 10^−3^					σ × 10^−10^				
M1	3.06	2.46	3.59	3.85	9.66	0.52	4.20	62.66	390.27	16,351.66
M2	4.61	4.01	4.31	3.85	11.21	0.79	7.02	62.66	666.43	19,468.31
M3	5.33	4.73	4.31	4.57	11.93	0.98	8.43	76.52	803.92	20,949.06
M4	6.54	5.94	5.52	5.78	13.14	1.20	10.72	99.06	803.92	23,171.24

**Table 5:** Electrical and critical resistivity for humidity-aged samples.

Immersion Time (h)	Electrical Resistivity (Ω × m) of M1	Critical Resistivity (Ω × m) of M1	Electrical Resistivity (Ω × m) of M2	Critical Resistivity (Ω × m) of M2	Electrical Resistivity (Ω × m) of M3	Critical Resistivity (Ω × m) of M3	Electrical Resistivity (Ω × m) of M4	Critical Resistivity (Ω × m) of M4
0	6.12 × 10^5^	1.83 × 10^5^	5.11 × 10^5^	1.53 × 10^5^	4.76 × 10^5^	1.43 × 10^5^	4.31 × 10^5^	1.29 × 10^5^
168	5.87 × 10^5^	1.83 × 10^5^	5.69 × 10^5^	1.53 × 10^5^	4.58 × 10^5^	1.43 × 10^5^	3.89 × 10^5^	1.29 × 10^5^
336	5.76 × 10^5^	1.83 × 10^5^	5.67 × 10^5^	1.53 × 10^5^	4.68 × 10^5^	1.43 × 10^5^	3.86 × 10^5^	1.29 × 10^5^
504	4.88 × 10^5^	1.83 × 10^5^	4.89 × 10^5^	1.53 × 10^5^	4.27 × 10^5^	1.43 × 10^5^	3.77 × 10^5^	1.29 × 10^5^
672	5.49 × 10^5^	1.83 × 10^5^	5.33 × 10^5^	1.53 × 10^5^	4.14 × 10^5^	1.43 × 10^5^	3.48 × 10^5^	1.29 × 10^5^
840	4.75 × 10^5^	1.83 × 10^5^	4.49 × 10^5^	1.53 × 10^5^	3.98 × 10^5^	1.43 × 10^5^	3.46 × 10^5^	1.29 × 10^5^

**Table 6:** Lifetime and remaining lifetime for the samples exposed to moisture.

Samples	Equation	Intercept = y	a	b	x = Lifetime (h)	Remaining Lifetime (h)
M1	y =−150.41x + 611107	183,467.64	−150.41	611,107	2843	2003
M2	y =−84.961x + 555326	153,395.10	−84.96	555,326	4731	3891
M3	y =−90.065x + 471894	142,811.71	−90.07	471,894	3654	2814
M4	y =−96.22x + 420010	129,282.65	−96.22	420,010	3021	2181

**Table 7:** Electrical and critical resistivity for temperature-aged samples.

Aging Time (h)	Electrical Resistivity (Ω × m) of M1	Critical Resistivity (Ω × m) of M1	Electrical Resistivity (Ω × m) of M2	Critical Resistivity (Ω × m) of M2	Electrical Resistivity (Ω × m) of M3	Critical Resistivity (Ω × m) of M3	Electrical Resistivity (Ω × m) of M4	Critical Resistivity (Ω × m) of M4
0	6.15 × 10^5^	1.85 × 10^5^	5.19 × 10^5^	1.56 × 10^5^	4.78 × 10^5^	1.44 × 10^5^	4.33 × 10^5^	1.30 × 10^5^
168	5.81 × 10^5^	1.85 × 10^5^	5.78 × 10^5^	1.56 × 10^5^	5.24 × 10^5^	1.44 × 10^5^	4.99 × 10^5^	1.30 × 10^5^
336	5.48 × 10^5^	1.85 × 10^5^	5.39 × 10^5^	1.56 × 10^5^	4.80 × 10^5^	1.44 × 10^5^	4.48 × 10^5^	1.30 × 10^5^
504	5.13 × 10^5^	1.85 × 10^5^	5.03 × 10^5^	1.56 × 10^5^	4.29 × 10^5^	1.44 × 10^5^	4.13 × 10^5^	1.30 × 10^5^
672	4.64 × 10^5^	1.85 × 10^5^	4.70 × 10^5^	1.56 × 10^5^	4.07 × 10^5^	1.44 × 10^5^	3.73 × 10^5^	1.30 × 10^5^
840	4.38 × 10^5^	1.85 × 10^5^	4.41 × 10^5^	1.56 × 10^5^	3.88 × 10^5^	1.44 × 10^5^	3.51 × 10^5^	1.30 × 10^5^

**Table 8:** Lifetime and remaining lifetime for the samples aged 840 h at 100 °C.

Samples	Equation	Intercept = y	a	b	x = Lifetime (h)	Remaining Lifetime (h)
M1	y = −215.8x + 617,219	184,614.16	−215.80	617,219	2005	1165
M2	y = −126.74x + 561,333	155,605.96	−126.74	561,333	3201	2361
M3	y = −145.03x + 512,173	143,531.69	−145.03	512,173	2542	1702
M4	y = −139.91x + 478,415	129,874.73	−139.91	478,415	2491	1651

**Table 9:** Mean ± SD values of indentation hardness (H_IT_), Vickers hardness (HV), and elastic modulus (E_IT_) for the unaged and aged samples exposed to humidity and high temperature (100 °C) for 840 h.

Samples	Mean H_IT_ ± SD (GPa)	Mean Vickers Hardness HV	Mean E_IT_ ± SD (GPa)
M1	0.035 ± 0.003	3.254 ± 0.290	0.625 ± 0.048
M2	0.042 ± 0.004	3.846 ± 0.388	0.732 ± 0.030
M3	0.038 ± 0.002	3.558 ± 0.187	0.696 ± 0.035
M4	0.037 ± 0.001	3.385 ± 0.114	0.698 ± 0.029
M1W	0.040 ± 0.002	3.670 ± 0.164	0.669 ± 0.030
M2W	0.042 ± 0.006	3.932 ± 0.542	0.706 ± 0.023
M3W	0.041 ± 0.005	3.797 ± 0.430	0.694 ± 0.065
M4W	0.040 ± 0.002	3.675 ± 0.190	0.691 ± 0.029
M1TT	0.072 ± 0.005	6.694 ± 0.504	1.411 ± 0.245
M2TT	0.085 ± 0.005	7.918 ± 0.475	1.871 ± 0.152
M3TT	0.082 ± 0.004	7.623 ± 0.337	1.785 ± 0.026
M4TT	0.079 ± 0.010	7.278 ± 0.899	1.730 ± 0.084

**Table 10:** Variation in mean values of indentation hardness (H_IT_), Vickers hardness (HV), and elastic modulus (E_IT_) of the unaged and aged composite samples relative to M1.

Mechanical Property	Increase Compared to M1 (%)										
	M2	M3	M4	M1W	M2W	M3W	M4W	M1TT	M2TT	M3TT	M4TT
H_IT_ (GPa)	16.67	7.89	5.41	12.50	16.67	14.63	12.50	51.39	58.82	57.32	55.70
Vickers hardness (HV)	14.62	10.20	10.46	6.58	11.47	9.94	9.55	55.71	66.60	64.99	63.87
E_IT_ (GPa)	15.39	8.54	3.87	11.34	17.24	14.30	11.46	51.39	58.90	57.31	55.29

**Table 11:** Water absorption of the samples M1–M4.

Samples/ Exposure Time	Water Absorption				
	168 h	336 h	504 h	672 h	840 h
M1	0.22	0.21	0.21	0.21	0.21
M2	0.16	0.12	0.16	0.16	0.19
M3	0.34	0.24	0.27	0.27	0.30
M4	0.33	0.36	0.35	0.35	0.38

## Data Availability

The data are included within the paper.
